# Indications of Endocrine Disruptor Effects of JP-5 Jet Fuel Using a Rat-Model Reproductive Study and an In Vitro Human Hormone Receptor Assay

**DOI:** 10.3390/toxics12030220

**Published:** 2024-03-16

**Authors:** William R. Howard, Joyce G. Rohan, Kimberly S. B. Yeager, Chester P. Gut, Kathleen A. Frondorf, Shawn M. McInturf, Nathan M. Gargas, Karen L. Mumy

**Affiliations:** 1 Naval Medical Research Unit Dayton, Wright-Patterson Air Force Base, Dayton, OH 45433, USAkaren.mumy@us.af.mil (K.L.M.); 2 Abbott Laboratories, Columbus, OH 43219, USA; 3 Reston Town Center, 1750 Presidents St, Reston, VA 20190, USA

**Keywords:** jet fuel, jet propellant, JP-5, JP-8, estrogen receptor, endocrine disruptor

## Abstract

Recent events concerning jet fuel contamination of drinking water have shown that we need a better understanding of the effects of ingested jet fuel. To this end, a reproductive study with ingested jet fuel in rats was undertaken with relatively high concentrations of Jet Propellant (JP)-5 along with a human estrogen receptor activation in vitro assay using JP-5, JP-8, and an alternative jet fuel derived from the camelina plant referred to as HydroRenewable Jet (HRJ) fuel, to help evaluate potential effects of ingested jet fuel. The results of the in vivo study provide evidence that JP-5 can act as an endocrine disruptor, with specific observations including altered hormone levels with JP-5 exposure (significantly lower estradiol levels in male rats and significantly increased Dehydroepiandrosterone levels in females), and a decreased male/female offspring ratio. The in vitro hormone receptor activation assay indicated that JP-5 and JP-8 are capable of upregulating human estrogen receptor (ER) activity, while HRJ was not active in the ER assay. The jet fuels were not able to activate androgen or glucocorticoid receptors in further in vitro assays. These results infer potential endocrine disruption associated with JP-5, with activation of the estrogen receptor as one potential mechanism of action.

## 1. Introduction

Exposure to jet fuels can occur through a variety of routes, with the most likely routes being dermal (skin) and inhalation (respiratory). Generally speaking, ingestion as a route of exposure for jet fuel has not been widely studied or considered a priority for toxicological studies, given that the more occupational routes have taken precedent due to their greater likelihood of occurrence. However, oral exposure can occur in situations where food or drinking water comes into contact with the fuel, such as may occur with spills or accidental leaks. Both types of these incidents have occurred in recent years, including the contamination of drinking water with Jet Propellant (JP)-5 aboard a U.S. aircraft carrier, the USS Nimitz, in September of 2022 [1,2] and a series of unfortunate leaks that culminated in the accidental release of as much as 14,000 gallons of JP-5 from the Navy’s Red Hill Fuel Storage Facility at Joint Base Pearl Harbor–Hickam (JBPHH) in late 2021 [3]. During the JBPHH incident, the residential water for ~93,000 Service Members, military families, employees, and the surrounding communities were impacted, with many residents voicing concerns over the potential short- and long-term health impacts of fuel consumption [3]. Without the appropriate studies to consult, there has been little scientific basis for addressing concerns regarding oral exposure or answering the health-related questions raised by these events, including whether jet fuels such as JP-5 can potentially impact hormone levels, reproduction, and/or development. 

Several studies have detailed cases of self-reported symptoms, which included dizziness, attention deficits, headache, and various cognitive impairments with exposure to JP-5 vapors [4,5,6,7]. In contrast, there are significantly fewer published studies reporting effects of exposure to jet fuel on other aspects of physiology, including endocrine function. A 2002 study reported that “low”-concentration occupational exposures of fuel and solvents (primarily JP-8) among female U.S. Air Force personnel resulted in adverse effects on endocrine markers associated with nonconceptive menstrual cycles, suggesting that jet fuel, or components thereof, can act as endocrine disruptors [8]. In support of this hypothesis, a 2015 publication suggested that benzene, toluene, ethylbenzene, and xylene, all four of which are constituents of jet fuels, may have endocrine disrupting properties at lower levels than expected [9]. Likewise, a 2018 study focusing on groundwater contamination with conventional oil and gas suggested potential impacts on endocrine bioactivities [10], while another previous study in male rats indicated that jet fuel can alter protein expression in rodent testes [11]. 

**Figure 1:**
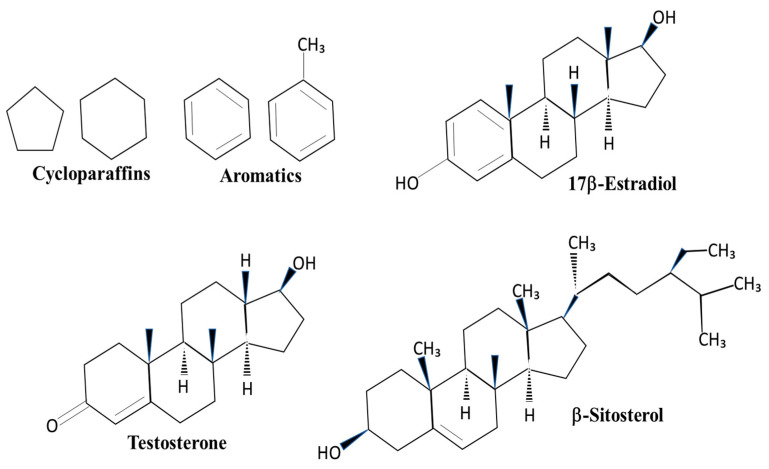
**Structures of cycloparaffins, aromatics, and sterol compounds.** Cycloparaffin and aromatic structures found in jet fuels are also components of sterol compounds. 17β-Estradiol is a steroid that activates the estrogen receptor. Testosterone is an activator of the androgen receptor. β-Sitosterol is a plant sterol found in the camelina plant.

The major components of JP-5 are approximately 53% straight-chain paraffins, 31% cycloparaffins, and 16% aromatics [12]. The significance of these structures related to endocrine disruption lies in the similarity of cycloparaffin and aromatic rings to endogenous steroids, such as estradiol and testosterone, as well as similarities to plant phytosterols, such as sitosterol (Figure 1). The structural similarities allow for the possibility that the components of JP-5 could mimic or compete with endogenous steroids in the body. By comparison, JP-8 has a higher percentage of straight-chain carbon structures than JP-5, approximately 72%, but nearly the same percentage of aromatics at approximately 16% [12]. HRJ is derived from the camelina plant, which is known to produce the phytoestrogen β-sitosterol, a known activator of the mammalian estrogen receptor [13], but it is unclear what constituents are in the HRJ jet fuel.

The current study used male and female Sprague Dawley rats and oral exposure to JP-5 to establish potential endocrine disruption and changes to reproductive health resulting from JP-5 ingestion. The occupational exposure setting of 5 days per week of exposure was chosen, and the maximum dose used was based on the study by Copper and Mattie (1996) using oral dosing of rats with JP-8 [14]. By using exposure periods that encompass a full spermatogenesis cycle for male rats and approximately four ovulatory cycles for females, this study was designed to detect impacts on reproductive health in adult rats. Further, observing fetal development in rats exposed to JP-5 prior to mating and throughout pregnancy allowed for insight into the developmental effects following JP-5 exposure. Additionally, an in vitro assay involving human estrogen, androgen, and glucocorticoid receptors in human cells provided insight into possible adverse effects due to ingestion of JP-5 while using JP-8 and HRJ as comparators.

## 2. Materials and Methods

### 2.1. Jet Fuels

Jet fuels were obtained through the U.S. Air Force Fuels and Energy Branch at Wright-Patterson Air Force Base. U.S. Navy jet fuel JP-5 (El Paso Corporation, Houston, TX, USA) was used for animal studies. U.S. Air Force jet fuel JP-8 (AGE Refining, San Antonio, TX, USA) and camelina plant-derived jet fuel (Bio-oil Derived Synthetic Paraffinic Kerosene, UPO LLC, Des Plaines, IL, USA) were used in the in vitro assays, as well as JP-5. 

### 2.2. Animal Study Design 

Sprague Dawley rats aged ~80 days were used in this study. Rats were maintained with a 12 h/12 h electronically controlled light/dark cycle and provided dry chow and water ad libitum in a temperature (20–26 °C)- and humidity (30–70%)-controlled vivarium. A blood sample was collected from all rats pre-exposure to use as a baseline for reproductive hormone levels. Food consumption and body weights were recorded daily during exposure for all animals and daily during gestation for bred females, and at a minimum of twice per week otherwise. 

Exposures were stagger-started in accordance with the reproductive physiology for each sex so that both sexes completed exposures at the same time to begin breeding. The rat spermatogenesis cycle is approximately 52 days in Sprague Dawley rats, with an additional 8.5 days required for epididymal transit; accordingly, male rats were exposed for 5 days/week for 9 weeks to encompass a complete spermatogenesis cycle with maturation of sperm in the epididymis. The rat ovulatory cycle is 4–5 days; accordingly, females were exposed for 5 days/week for 3 weeks to encompass approximately 4 ovulatory cycles prior to either breeding or euthanasia and necropsy, with exposures to jet fuel continuing through pregnancy. 

### 2.3. Exposure Groups

This study incorporated two sets of female rats, with one group exposed for the collection of ovulatory cycle data during the last week of exposure before being euthanized and ovaries collected at necropsy for histopathological examination. The second set of female rats was used for breeding and gestation, with euthanasia at gestation day (GD) 20 in order to examine the physical characteristics of the fetuses. Male rats were euthanized after mating to collect the testes for sperm analysis and histopathological examination. Numbers of animals available for evaluation are noted in the figures for each experiment.

### 2.4. Oral Administration

Animals were exposed via gavage with a 20-gauge, 3-inch curved stainless steel cannula ball-tipped gavage needle affixed to a glass syringe. Appropriate dose volumes were drawn into the syringe and the excess liquid was wiped from the stainless-steel gavage needle prior to administration. Control rats were administered 2.0 mL/kg of body weight with distilled water. JP-5 “low”-dose rats were administered 750 mg/kg of neat JP-5 at a volume of 0.86 mL/kg of body weight. JP-5 “high”-dose rats were administered 2000 mg/kg of neat JP-5 at a volume of 2.31 mL/kg of body weight. Dose volumes were based on a calculated fuel density of JP-5 at 868 mg/mL. Male rats were exposed for 5 days/week for 9 weeks and through mating. Female rats were exposed for 5 days/week for 3 weeks and, for animals that were bred, throughout pregnancy to gestational day 20.

### 2.5. Vaginal Cytology

During the final 7 days of exposure, including 5 days of gavage, vaginal lavage and cytology were used to assess ovulatory cycles. Using a pipette, approximately 20–25 µL of sterile 0.9% physiological saline was used to perform the lavage. The pipette tip was inserted approximately 3–5 mm in the vaginal opening, the fluid was expelled into the vagina and then flushed back and forth into the vagina 2–3 times, finally to be re-collected using the pipette. The lavage liquid was placed into an appropriate square on a labeled microscope slide and allowed to air-dry for at least 24 h prior to staining. Slides were stained with 0.1% methyl blue for approximately 1 min and de-stained in distilled water for approximately 1–2 min. The slides were viewed under a microscope at 10x–40x magnification to assess the estrous cycle stage. The predominance of any one specific type of cell was considered to be representative of a stage of the estrous cycle. Proestrus was indicated by a predominance of nucleated round epithelial cells. Estrus was indicated by a predominance of irregularly shaped cornified epithelial cells in which the nucleus is not well-defined or absent. Metestrus was indicated by an approximately equal distribution of leukocytes, cornified epithelial cells, and nucleated round epithelial cells. Diestrus was indicated by a predominance of leukocytes. A cycle was considered abnormal if there were three or more days in a row in the estrus phase; a cycle was also considered abnormal with four or more days of diestrus in a row.

### 2.6. Hormone Analyses

Blood samples were collected from each rat prior to the onset of exposure and at the time of necropsy. Reproductive hormone levels were assessed using a multi-array immunoassay kit (Meso Scale Discovery, Rockville, MD, USA) that quantified the levels of estradiol, progesterone, testosterone, and dehydroepiandrosterone (DHEA) simultaneously in one sample. These measurements were conducted in triplicate and data with a coefficient of variation (CV) greater than 30 were omitted.

### 2.7. Mating Procedure

Males and females were paired (25 pairs per exposure group were targeted, but due to attrition, actual numbers were 25 for the control group, 24 for the JP-5 750 mg/kg group, and 18 for the 2000 mg/kg group) for cohabitation until visible evidence of mating in the form of a vaginal plug was observed. Exposures to jet fuel continued until mating. Following successful mating, males from each pair were euthanized for sperm analysis. 

### 2.8. Sperm Analysis and Testicular Histology

One testis and one epididymis from each male were used for the assessment of sperm motility, density, morphology, and calculated average spermatid head count. The remaining testes and epididymes were used for histological examination of the seminiferous tubules. 

Sperm Count: Testes were removed and weighed. The capsule was removed, and the remaining testicle was placed into a round-bottom tube. A total of 3 volumes of homogenizing solution (0.9% saline, 0.5% Triton X100) per weight of testicle was added. The testicle was homogenized using a tissue tearer. Following homogenization, 100 µL of distilled water and 100 µL of homogenate was added to a microtube containing IDENT stain (Hamilton Thorne, Beverly, MA, USA). Samples were loaded into 2XCEL slides, cover-slipped, and analyzed on the Hamilton Thorne TOX IVOS computer-assisted sperm analyzer system (Hamilton Thorne, Beverly, MA, USA).

Sperm Motility: The epididymis was removed and weighed. Immediately, the epididymis was added to 10 mL of freshly prepared M-199 medium plus 0.5% bovine serum albumin (BSA) in a Petri dish. Using a #11 scalpel, the distal cauda of the epididymis was punctured approximately 10–15 times (avoiding blood vessels) to allow spermatozoa to enter the medium with care taken to ensure the spermatozoa avoided air contact. The dish was covered and placed on a 37 °C hotplate for approximately 1 min. Following warming, the tissue was removed, and the medium was gently swirled. The, 12 µL of the medium was pipetted from the dish onto a slide and the slide was analyzed on a Hamilton Thorne TOX IVOS computer-assisted sperm analyzer system (Hamilton Thorne, Beverly, MA, USA).

Sperm Morphology: The epididymis was removed and weighed. Immediately, the epididymis was added to 10 mL of freshly prepared M-199 medium plus 0.5% BSA in a Petri dish. Using a #11 scalpel, the distal cauda of the epididymis was punctured approximately 10–15 times (avoiding blood vessels) to allow spermatozoa to enter the medium. Care was taken to ensure the spermatozoa avoided air contact. The dish was covered and placed on a 37 °C hotplate for approximately 1 min. Following warming, the tissue was removed, and the medium was gently swirled. Then, 1 mL of the medium was pipetted into a tube containing 100 µL of formalin. Samples were refrigerated at 4 °C until analyzed. At time of analysis, 100 µL of the medium sample and formalin mixture was added to a microtube containing IDENT stain (Hamilton Thorne, Beverly, MA, USA), vortexed, and allowed to rest for 2 min. Then, 15 µL of the sample was pipetted onto a microscope slide and cover-slipped and the slide was analyzed on a Hamilton Thorne TOX IVOS computer-assisted sperm analyzer system (Hamilton Thorne, Beverly, MA, USA).

### 2.9. Pregnancy and Offspring Assessments

Bred females were maintained until gestation day 20, at which time they were humanely euthanized with carbon dioxide inhalation in conjunction with pneumothorax. Females then underwent necropsy and laparohysterectomy. The number of corpora lutea in each ovary was determined. The uterus was examined to determine the number of implantations, number of viable/non-viable fetuses, and number of implantation scars. Fetal position in the uterus and fetal body weights were recorded, and examinations were made for obvious external malformations or variations. An external examination of all fetuses (890 total) was performed, with anogenital distance measured to assess external sexual characteristics. Fetuses were euthanized by intraperitoneal (i.p.) injection of pentobarbital. Fetuses were necropsied to locate the gonads to determine the internal sex of the fetuses for comparison with external sexual characteristics for concordance. 

### 2.10. Mammalian Cell Co-Transfection Assays

JP-5 was used in an in vitro assay to evaluate its ability to activate the human estrogen, androgen, and/or glucocorticoid receptor. Another conventional jet fuel, JP-8, was used for comparison, as was an alternative jet fuel, camelina plant-derived Bio-oil-Derived Synthetic Paraffinic Kerosene (HydroRenewable Jet; HRJ). Mammalian cell co-transfection assays were performed as previously described [15,16,17]. Briefly, a eukaryotic expression plasmid for the expression of the appropriate nuclear receptor (pRShER for the expression of human estrogen receptor-α, pRShAR for the expression of human androgen receptor, or pRShGR for the expression of human glucocorticoid receptor) along with a luciferase reporter plasmid containing the appropriate receptor response element for the particular receptor being tested [18,19] were co-transfected into human embryonic kidney 293 (HEK293) cells by calcium phosphate co-precipitation. A constitutively expressed pRSV-β-galactosidase plasmid was included in all transfections for the normalization of luciferase data. Transfected cells were grown in DMEM/F12 medium containing 10% charcoal-stripped fetal calf serum and specific test sterols. To test the ability of jet fuels to modulate human receptor activity, JP-8, JP-5, or HRJ (1:1 by volume) were tested at concentrations of 0, 0.001, 0.05, 0.1, 0.5, 1, 5, and 10 mg/mL. After 48 h, cells were lysed and assayed (following the addition of luciferin) for luciferase activity on a luminometer and for β-galactosidase (following the addition of ONPG) at 415 nm on a microplate reader. The luciferase response was normalized to the β-galactosidase rate. Controls included assays without the co-transfected steroid receptor plasmid for all compounds tested. All results shown were dependent on the presence of the nuclear receptor and test compounds as well as on the presence of the plasmid with the appropriate response element for the nuclear receptor being tested. All experiments were performed in triplicate, and the readings for each separate experiment were made in triplicate. Test steroids were obtained from commercially available sources. Stock solutions of fuels were made based on the density of the particular jet fuel (845 mg/mL for JP-8, 868 mg/mL for JP-5, and 794 mg/mL for HRJ) with serial dilutions in ethanol of 10, 100, and 1000 times to facilitate reaching the concentrations listed above.

### 2.11. Statistical Analyses

All data analyses were performed in Microsoft Excel and Sigmaplot v.13.0. Data that passed normality and contained more than two groups were analyzed using a one-way analysis of variance (ANOVA) in Sigmaplot v.13.0. Multiple comparison analyses following significant one-way ANOVAs were conducted using the Holm–Sidak method. Data that did not pass normality and contained more than two groups were analyzed using the non-parametric Kruskal–Wallis one-way ANOVA in Sigmaplot v.13.0. Multiple comparison analyses following significant Kruskal–Wallis one-way ANOVAs were conducted using Dunn’s method. Data containing only two groups were analyzed in Microsoft Excel, using the two-tailed unpaired or paired *t*-test if normality was passed or the non-parametric Mann–Whitney if normality was not passed. A binomial distribution analysis was used to evaluate the male-to-female ratios of offspring, with the assumption that the distribution should be 50% based on the fact that spermatogenesis is a meiotic process that will produce 50% Y and 50% X chromosome-bearing spermatids.

## 3. Results 

### 3.1. Effects of JP-5 Exposure on Body Weight and Food Intake

**Figure 2:**
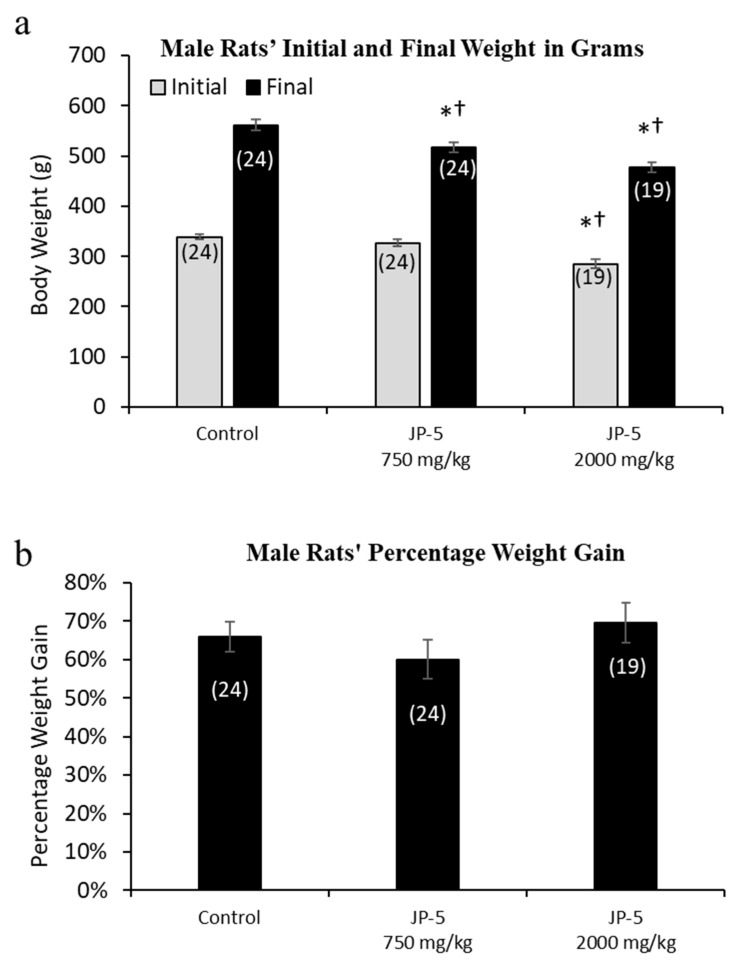
**Mean weight gain for male rats with JP-5 exposure.** Male rats were exposed by oral gavage to JP-5 for 9 weeks prior to being paired for mating, with exposures continuing until successful mating. (**a**) Although rats were randomly assigned to experimental groups, the initial mean body weight for the JP-5 2000 mg/kg body weight exposure group (gray bar) had an initial weight average that was significantly lower than both the control group and the 750 mg/kg exposure group (*† *p* < 0.001). The JP-5 750 mg/kg body weight exposure group had a final average body weight (black bar) that was significantly lower than the control group (* *p* = 0.007), even though there was no initial statistically significant difference between these groups. The JP-5 at 2000 mg/kg final body weight group (black bar) had final average weights that remained significantly lower than both the control (* *p* < 0.001) and 750 mg/kg body weight groups († *p* < 0.010). (**b**) There were no significant differences between groups when analyzed as mean percentage weight gain over the course of the study. Numbers in parentheses represent n values. Data presented as mean +/− standard error of the mean (SEM).

Rats were randomly assigned to exposure groups and were monitored for body weight changes and food intake during jet fuel exposure. No rats lost weight over the course of the study, suggesting that the jet fuel exposures were not associated with significant digestive problems that would have otherwise led to weight loss. The final weight for the male rats appeared to be slightly, but significantly, dose-dependently affected by JP-5 exposure (Figure 2a). The analysis of the male final weights using the one-way ANOVA revealed a significant difference across groups, with F(2, 64) = 15.136, *p* < 0.001 (n = 19–24), and the multiple comparison using the Holm–Sidak method yielded a significant reduction in the weight of rats in the JP-5 2000 mg/kg group compared to the control (t = 5.479, *p* < 0.001) and also in the JP-5 750 mg/kg group compared to the control (t = 3.019, *p* = 0.007). Rats in the JP-5 2000 mg/kg group had reduced weight compared to rats in the JP-5 750 mg/kg group, with t = 2.641, *p* = 0.010. However, further analysis of pre-exposure weights also revealed a significant difference across groups (F(2, 64) = 15.019, *p* < 0.001), with rats in the JP-5 2000 mg/kg group having significantly smaller body weights compared to the control animals (t = 5.298, *p* < 0.001) or the JP-5 Low group (t = 4.121, *p* < 0.001). 

Because of the significant difference in the pre-exposure weights of rats in the JP-5 2000 mg/kg group, we evaluated the body weight data as percentage weight gained over the course of the study. When normalized as percentage weight gained over the course of the study, there were no differences noted for weight gain in male rats in this study (*p* > 0.05, one-way ANOVA and Kruskal–Wallis) (Figure 2b). 

**Figure 3:**
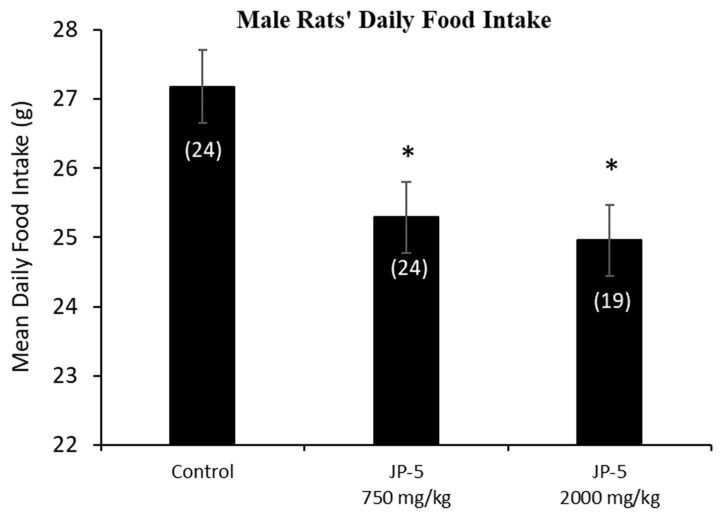
**Male rats’ daily food intake during 9 weeks of JP-5 exposure.** Mean daily food intake in grams was significantly lower for both JP-5 750 mg/kg group (* *p* = 0.028) and JP-5 2K mg/kg group (* *p* = 0.021) when compared to control rats. Numbers in parentheses represent n values. Data presented as mean *+/−* SEM.

The mean daily food intake was significantly lower for male rats in the JP-5 750 mg/kg group (t = 2.527, *p* = 0.028) and JP-5 2000 mg/kg group (t = 2.786, *p* = 0.021) compared to food consumed by rats in the control group. (Figure 3). The significantly lower daily food intake noted in Figure 3 for both JP-5 exposure groups directly corresponds to the significantly lower body weights for the same exposure groups shown in Figure 2a. 

**Figure 4:**
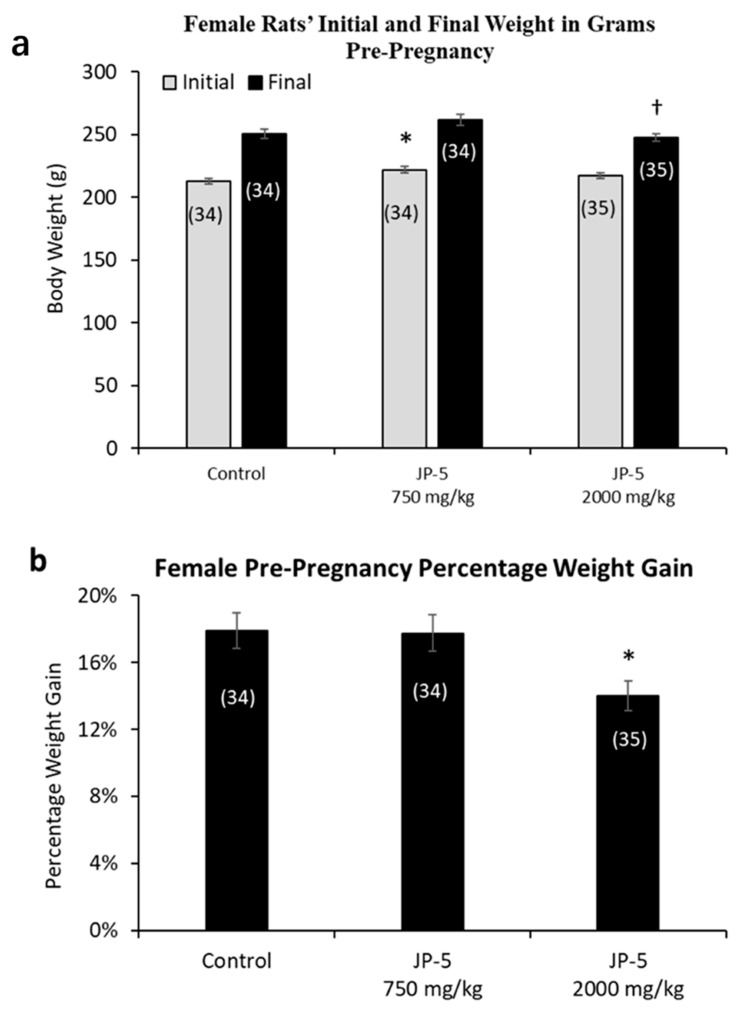
**Mean weight gain for female rats with JP-5 exposure pre-pregnancy.** Female rats were exposed by oral gavage to jet fuels for 3 weeks prior to mating. Mean weight gain pre-pregnancy was calculated for initial weight and final weight before mating. (**a**) Animals were randomly assigned to groups for the procedure, with the JP-5 at 750 mg/kg body weight group having initial mean body weights (gray bars) significantly higher than the control group (* *p* = 0.016). Final post-exposure weight data (black bars) from female rats in the JP-5 2K mg/kg group were significantly lower compared to weight data from female rats in the JP-5 750 mg/kg group († *p* = 0.028). (**b**) Percentage weight gain was significantly lower in rats exposed to JP-5 at 2000 mg/kg body weight compared to both control (* *p* = 0.022) and 750 mg/kg body weight groups († *p* = 0.02). Numbers in parentheses represent n values. Error values = standard error of the mean.

Female rats were randomly assigned to either the control, JP-5 750 mg/kg, or JP-5 2000 mg/kg exposure groups with three weeks of exposure before either pre-mating or ovulatory assessments as described in the Materials and Methods. The random assignment of animals to the different groups resulted in a significantly higher initial weight for the female rats in the JP-5 750 mg/kg group when compared to the control group (t = 2.857, *p* = 0.016, n = 34–35 Holm–Sidak method, following significant one-way ANOVA with F(2, 100) = 4.083, *p* = 0.02) (Figure 4a, gray bars). The analysis of the female post-exposure, pre-pregnancy weight data with the one-way ANOVA revealed significant differences across groups, with F(2, 100) = 3.850, *p* = 0.025, and multiple comparison using the Holm–Sidak method yielded significantly reduced final weights of female rats in the JP-5 2000 mg/kg group when compared to female rats in the JP-5 750 mg/kg group (t = 2.651, *p* = 0.028) (Figure 4a, black bars). The final, post-exposure weight data were also normalized to the initial, pre-exposure values to measure percent of weight gained. Analysis of the post-exposure, pre-pregnancy weight gain data using a one-way ANOVA revealed significant difference across groups, with F(2, 100) = 4.817, *p* = 0.01. Multiple comparison using the Holm–Sidak method yielded significantly less weight gain in female rats in the JP-5 2000 mg/kg group compared to the control (t = 2.730, *p* = 0.022) or compared to the JP-5 750 mg/kg group (t = 2.630, *p* = 0.020) (Figure 4b). The weight gain of female rats in the JP-5 750 mg/kg group was not statistically different from the control females (t = 0.010, *p* = 0.921).

**Figure 5:**
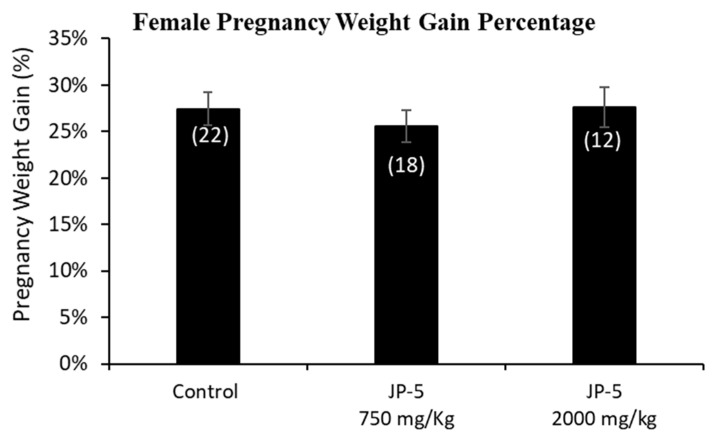
**Mean weight gain for female rats with JP-5 exposure during pregnancy.** Female rats were exposed by oral gavage to jet fuels during pregnancy. Mean weight gain for pregnancy was calculated for the final pre-pregnancy weight and the weight of the rats after 14 days of pregnancy. Weights at gestation day 14 were normalized to the respective pre-pregnancy weights to assess weight gain. There was no statistically significant effect on pregnancy weight gain resulting from JP-5 exposure (*p* = 0.687). Numbers in parentheses represent n values. Data presented as mean *+/−* SEM.

Only a subset of female rats were bred and became pregnant. The pregnancy weights were evaluated as the weight gained on GD 14, a timeframe that allows for a reasonable reckoning of differences among the exposure groups during pregnancy, normalized to the respective pre-pregnancy weight (Figure 5). The pregnancy weight gain data passed normality and was analyzed using a one-way ANOVA, yielding no statistical differences among groups, with F(2, 49) = 0.378, *p* = 0.687, n = 12–22 (Figure 5). 

**Figure 6:**
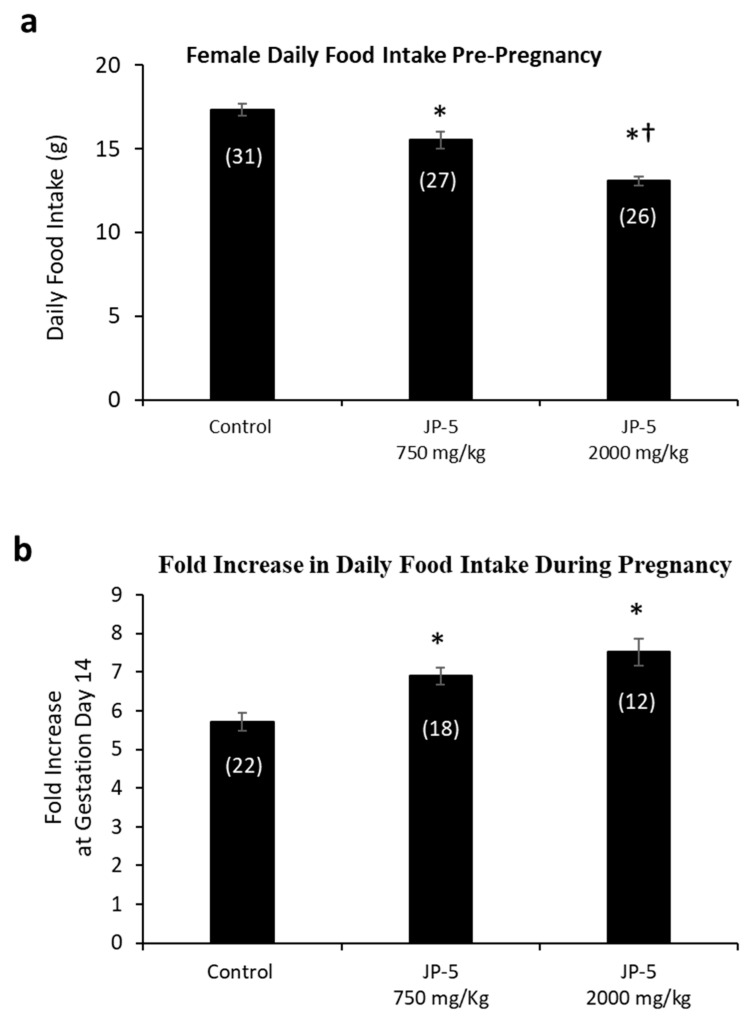
**Daily food intake for female rats both pre-pregnancy and during pregnancy.** (**a**) Mean daily food intake in grams prior to mating was significantly lower for the JP-5 Low group compared to the control group (* *p* < 0.001). Female rats in the JP-5 High group showed mean daily food intake that was significantly lower than either the control or the JP-5 Low groups (*† *p* < 0.001). (**b**) The fold increase in food intake during pregnancy was significantly higher for the JP-5 750 mg/kg group (* *p* = 0.002) and the JP-5 2K mg/kg group (* *p* < 0.001) when compared to the control group. Numbers in parentheses represent n values. Data presented as mean *+/−* SEM.

The results showed a dose-dependent increase in the average daily food intake of female rats resulting from JP-5 exposure (Figure 6a). Pre-pregnancy, post-exposure food intake data for female rats were reduced following JP-5 exposure, as analyzed using a one-way ANOVA, revealing significant differences across groups, with F(2, 81) = 30.139, *p* < 0.001, n = 26–31. Reduced daily food intake was observed for the JP-5 750 mg/kg group compared to the control (t = 3.354, *p* < 0.001, Holm–Sidak comparison), and for the JP-5 2000 mg/kg group compared to the control (t = 7.760, *p* < 0.001, Holm–Sidak comparison). The food intake from female rats in the JP-5 2000 mg/kg group was also significantly less than food intake from female rats in the JP-5 750 mg/kg group (t = 4.297, *p* < 0.001). 

A subset of female rats exposed to JP-5 750 mg/kg or JP-5 2000 mg/kg that became pregnant had increased food consumption during pregnancy relative to their food intake pre-pregnancy, as measured on GD 14 (Figure 6b). There was statistically significant difference across groups, with F(2, 49) = 12.586, *p* < 0.001, n = 12–22. Multiple comparison using the Holm–Sidak method yielded significantly higher food intake from pregnant rats in the JP-5 750 mg/kg group compared to the control (t = 3.483, *p* = 0.002) and also from pregnant rats in the JP-5 2000 mg/kg group compared to the control (t = 4.700, *p* < 0.001). 

### 3.2. JP-5 Effects on Male Rat Reproductive Organ Weights, Sperm Production, and Sperm Motility

**Figure 7:**
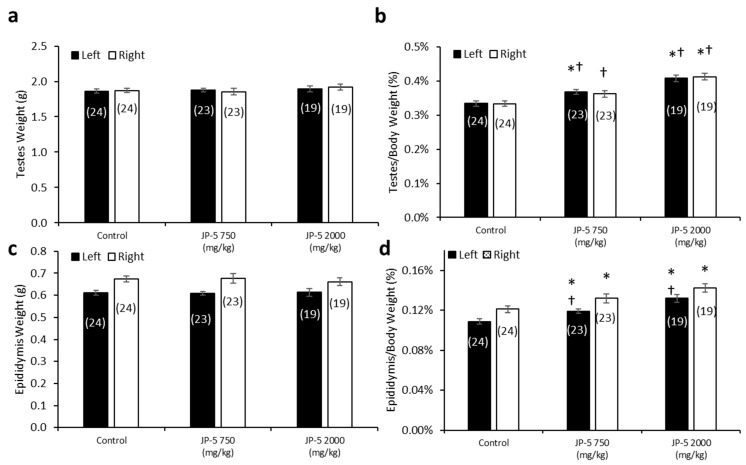
**Testis and epididymis weights in male rats exposed to jet fuel via gavage**. (**a**) There were no significant differences found in absolute testis weight among groups. (**b**) When mean testis weights were normalized to body weights, there was a significant difference across experimental groups. Normalized left testis weights from rats in the JP-5 Low group were significantly larger than those of rats in the control group (* *p* = 0.005). Normalized left and right testis weights from rats in the JP-5 High group were significantly larger than those from rats in the control (* *p* < 0.001) or JP-5 Low groups (^†^ *p* = 0.005 (left testis), ^†^ *p* = 0.018 (right testis)). (**c**) There were no significant differences found in absolute weights of epididymis in rats treated following JP-5 exposure via oral gavage. (**d**) When epididymis weights were normalized to body weights, there was a significant difference across experimental groups. There was a significant increase in epididymis weight relative to body weight for rats exposed to JP-5 Low compared to control rats (* *p* = 0.018 (left), * *p* = 0.049 (right)). Rats exposed to JP-5 High had greater mean percentage of left epididymis weight to body weight that was significantly higher than the averages for both control (* *p* < 0.001) and JP-5 Low groups (^†^ *p* = 0.005), while the normalized right epididymis weight values were significantly higher than the control group (* *p* < 0.001). Numbers in parentheses represent n values. Data presented as means *+/−* SEM.

There were no significant differences in the absolute mean of the left and right testis weights across the exposure groups (*p* > 0.05, one-way ANOVA and Kruskal–Wallis, n = 19–24) (Figure 7a). However, there were dose-dependent effects of JP-5 exposure on testis weight when normalized to body weight, with both the JP-5 750 mg/kg and JP-5 2000 mg/kg exposure groups having a higher normalized testis weight than the control group (Figure 7b). The one-way ANOVA of the normalized left testis weight data resulted in F(2, 63) = 19.900, *p* < 0.001, and a multiple comparison analysis using the Holm–Sidak method yielded significant differences between the control and JP-5 2000 mg/kg groups (t = 6.309, *p* < 0.001), the control and JP-5 750 mg/kg groups (t = 2.908, *p* = 0.005), and between the JP-5 2000 mg/kg and JP-5 750 mg/kg groups (t = 3.512, *p* = 0.002). The normalized right testis weight data failed normality, and thus, a Kruskal–Wallis one-way ANOVA was used to assess statistical significance, resulting in H = 23.732 (df = 2), *p* < 0.001. Multiple comparison of the normalized right testis weight data was performed using Dunn’s method and yielded significant differences between the JP-5 2000 mg/kg and control groups (Q = 4.871, *p* < 0.001), and between the JP-5 2000 mg/kg and JP-5 750 mg/kg groups (Q = 2.744, *p* = 0.018). There was a trending increase in the weight of the right testis in the JP-5 750 mg/kg group compared to the control; however, this was not statistically significant, with Q = 2.212, *p* = 0.08.

There were no significant differences in the absolute mean left and right epididymis weights across the groups (*p* > 0.05, one-way ANOVA and Kruskal–Wallis, n = 19–24) (Figure 7c). However, there was a dose-dependent increase in epididymis weights when normalized to the respective body weights resulting from JP-5 exposure (Figure 7d). The one-way ANOVA of the normalized left epididymis weight data resulted in F(2, 63) = 15.002, *p* < 0.001, and multiple comparison analysis using the Holm–Sidak method yielded significant differences between the control and JP-5 2000 mg/kg groups (t = 5.476, *p* < 0.001), the control and JP-5 750 mg/kg groups (t = 2.437, *p* = 0.018), and between the JP-5 2000 mg/kg and JP-5 750 mg/kg groups (t = 3.131, *p* = 0.005). The normalized right epididymis weight data failed normality, and thus, a Kruskal–Wallis one-way ANOVA was used to assess statistical significance, resulting in H = 15.956 (df = 2), *p* < 0.001. Multiple comparison of the normalized right epididymis weight data using Dunn’s method yielded a significant difference between the JP-5 2000 mg/kg and control groups (Q = 3.943, *p* < 0.001), and between the JP-5 Low and control groups (Q = 2.399, *p* = 0.049). Comparison between the JP-5 750 mg/kg and JP-5 2000 mg/kg exposure groups yielded Q = 1.647, *p* = 0.299. 

Taking into consideration that the rats used in this study were already mature, the fact that the absolute weights of the testes and epididymis are not statistically different across the groups is an indication that the test article did not exert a toxic influence on these already developed organs. It should also be noted that the rats with the largest normalized organ-to-body weight ratios (Figure 7b,d for testis and epididymis, respectively) were both of the JP-5-exposed groups that also showed the lowest absolute body weight (Figure 2a) and the lowest average daily food intake (Figure 3), which lends support to the supposition that these observations are most likely attributable to the JP-5 exposures upsetting digestion and potentially suppressing appetite. 

**Figure 8:**
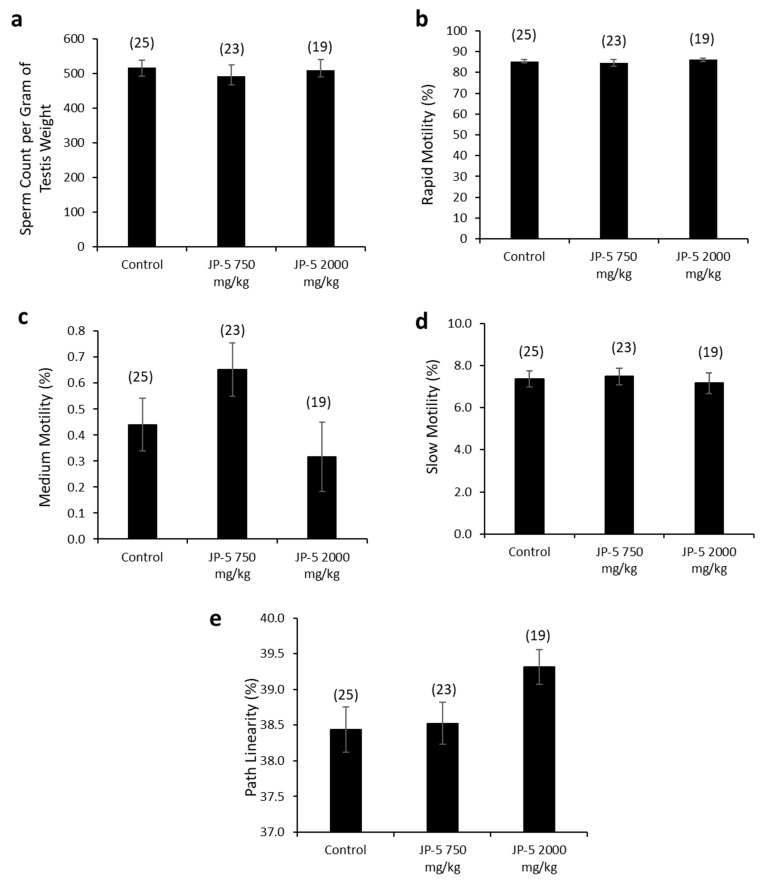
**Effects of JP-5 exposure on sperm production and motility.** (**a**) Amount of sperm produced per gram of testis was not significantly different across groups (*p* > 0.05, one-way ANOVA, n = 19–25). (**b**) Percent of sperm with rapid motility did not show any statistically significant differences among groups (*p* > 0.05, Kruskal–Wallis one-way ANOVA). (**c**) Percent of sperm with medium motility did not show any statistically significant differences among groups (*p* > 0.05, Kruskal–Wallis one-way ANOVA). (**d**) Percent of sperm with slow motility did not show any statistically significant differences among groups (*p* > 0.05, Kruskal–Wallis one-way ANOVA). (**e**) There was a trending increase in percent of sperm showing pathway linearity from male rats in the JP-5 High group (*p* = 0.053, one way ANOVA, n = 19–25). Numbers in parentheses represent n values. Data presented as mean *+/−* SEM.

There were no apparent effects of JP-5 exposures on sperm production measured per gram of testis mass (data passed normality, F(2, 65) = 0.243, *p* = 0.785, n = 19–25, one-way ANOVA) (Figure 8a). Sperm motility showed no adverse effects in either of the JP-5 exposure groups, with no significant differences from controls for percent rapid motility (data failed normality, H = 0.445, df = 2, *p* = 0.801, n = 19–25, Kruskal–Wallis) (Figure 8b), percent medium motility (data failed normality, H = 5.378, df = 2, *p* = 0.068, n = 19–25, Kruskal–Wallis) (Figure 8c), or percent slow motility (data failed normality, H = 0.547, df = 2, *p* = 0.761, n = 19–25, Kruskal–Wallis) (Figure 8c). 

There was a trending increase in sperm path linearity from rats in the JP-5 2000 mg/kg group compared to the control (Figure 8e). The data failed normality, and the Kruskal–Wallis one-way ANOVA yielded H = 5.862 (df = 2), *p* = 0.053 (n = 19–25). A direct comparison between the control and the JP-5 2000 mg/kg group data using a two-tailed, unpaired *t*-test yielded *p* = 0.04. However, a direct comparison using the non-parametric Mann–Whitney Rank Sum Test yielded *p* = 0.065. 

Other sperm traits analyzed included path velocity, progressive velocity, track speed, lateral amplitude, beat frequency, straightness, linearity and elongation, and percent static, with no statistically significant differences or trends to lend to interpretation for the difference in path linearity for sperm exposed to the higher concentration of JP-5. There were also no adverse effects due to JP-5 exposure on sperm morphology. 

### 3.3. Hormonal Effects on Male Rats

**Figure 9:**
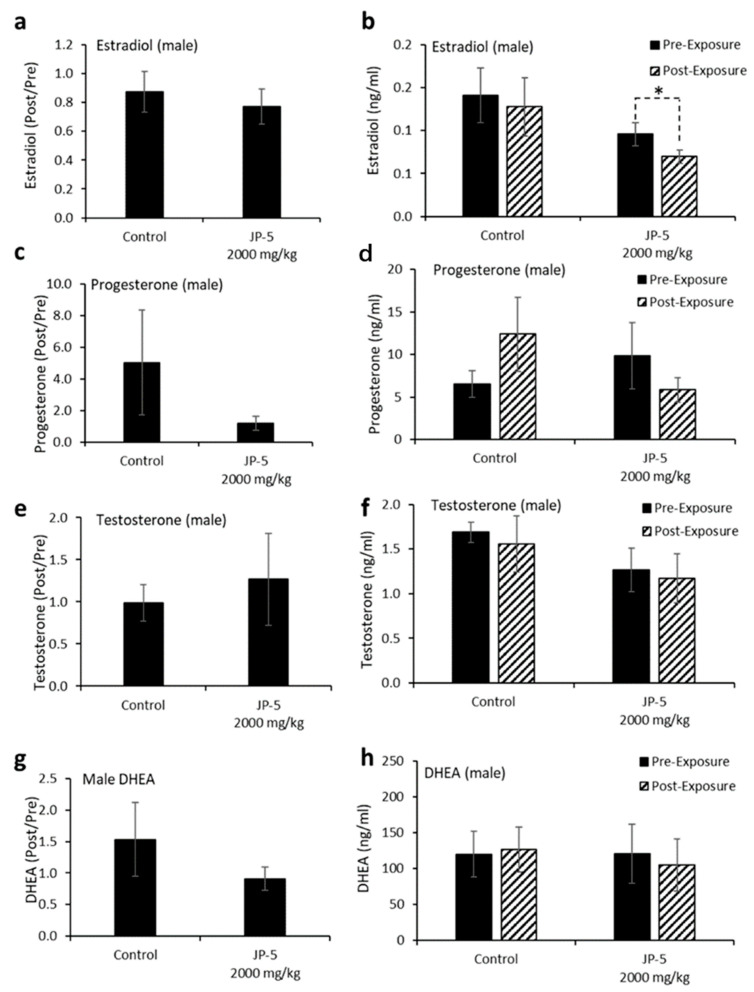
**Hormone levels for male rats following JP-5 exposure.** Hormone levels were measured as described in Methods. Jet fuel used was JP-5 at either 750 or 2000 mg/Kg body weight. (**a**) Estradiol levels at post-exposure were normalized to their respective pre-exposure levels. There were no statistically significant differences vs. control for JP-5 exposure (*p* = 0.45, unpaired *t*-test, n = 8 control, n = 7 JP-5 High). (**b**) Absolute estradiol levels were plotted before exposure (black bar) and after exposure (patterned bar). Estradiol levels were decreased at post-exposure compared to pre-exposure levels for JP-5-exposed rats (* *p* = 0.028, paired *t*-test, n = 8 control, n = 7 JP-5 High). (**c**) Progesterone levels at post-exposure were normalized to their respective pre-exposure levels. There were no statistically significant differences vs. control for JP-5 exposure (*p* = 0.36, unpaired *t*-test, n = 7). (**d**) Absolute progesterone levels plotted before exposure (black bar) and after exposure (patterned bar). Trending increase in post-exposure progesterone levels compared to pre-exposure levels in control rats (*p* = 0.29, paired *t*-test, n = 7). Trending reduction in post-exposure progesterone levels compared to pre-exposure levels in rats exposed to JP-5 (*p* = 0.38, paired *t*-test, n = 7). (**e**,**f**) There were no significant differences in the levels of testosterone between the control and JP-5 group when normalized to the respective pre-exposure levels (*p* = 0.65, unpaired *t*-test, n = 7) or when compared to their pre-exposure values within the control and JP-5 group (*p* = 0.64, paired *t*-test, n = 7). (**g**,**h**) There were no significant differences in the levels of DHEA between the control and JP-5 group when normalized to the respective pre-exposure levels (*p* = 0.91, unpaired *t*-test, n = 9 control, n = 5 JP-5 High) or when compared to their pre-exposure values within the control and JP-5 group (*p* = 0.86, paired *t*-test, n = 9 control, n = 5 JP-5 High).

Levels of hormones were evaluated for a subset of males following experimental exposures and mating as described in the Materials and Methods. Only control rats and rats exposed to 2000 mg of JP-5/kg were included in these evaluations. There were no significant reductions in the estradiol level in male rats compared to the control when data were analyzed as ratios of post- to pre-exposure values (*p* = 0.45, two-tailed, unpaired *t*-test, n = 8 control, n = 7 JP-5 2000 mg/kg group) (Figure 9a). However, a comparison of pre-exposure values with post-exposure values within the JP-5 2000 mg/kg group showed a significant reduction in estradiol levels (*p* = 0.028, two-tailed paired *t*-test), a difference that was not present in the control group (*p* = 0.5) (Figure 9b). 

There were no statistically significant differences in progesterone post-to-pre-exposure ratios noted (*p* = 0.36, two-tailed, unpaired *t*-test, n = 7) (Figure 9c), but the data did show that while control rats had a trending increase (*p* = 0.29) in progesterone levels following mating, the JP-5-exposed rats had a trending decrease (*p* = 0.38) in progesterone at the conclusion of the experimental procedures (Figure 9d). 

There were no significant differences observed in testosterone levels when data were analyzed as post-to-pre-exposure ratios (*p* = 0.65, two-tailed, unpaired *t*-test, n = 7) (Figure 9e) or when post-exposure values were statistically compared to pre-exposure values for the control and JP-5 2000 mg/kg groups (*p* = 0.64, 0.79, two-tailed, paired *t*-test) (Figure 9f). 

Similarly, there were no significant differences observed in DHEA levels when data were analyzed as post-to-pre-exposure ratios (*p* = 0.91, two-tailed, unpaired *t*-test, n = 9 control, n = 5 JP-5 2000 mg/kg) (Figure 9g) or when post-exposure values were statistically compared to pre-exposure values for the control and JP-5 2000 mg/kg groups (*p* = 0.86, 0.39, two-tailed, paired *t*-test) (Figure 9h). 

### 3.4. Effects on Female Rats

**Figure 10:**
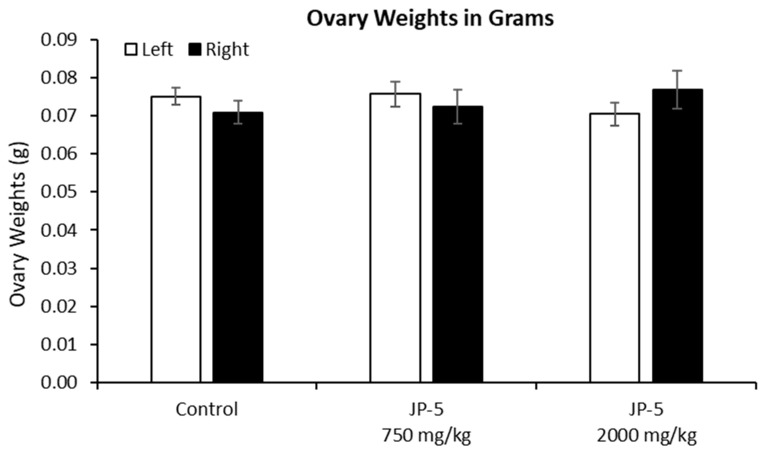
**Ovary weights for females after JP-5 exposure.** There were no significant differences found in the absolute weights of ovaries across the treatment groups (*p* = 0.371 for left, *p* = 0.596 for right ovary).

**Figure 11:**
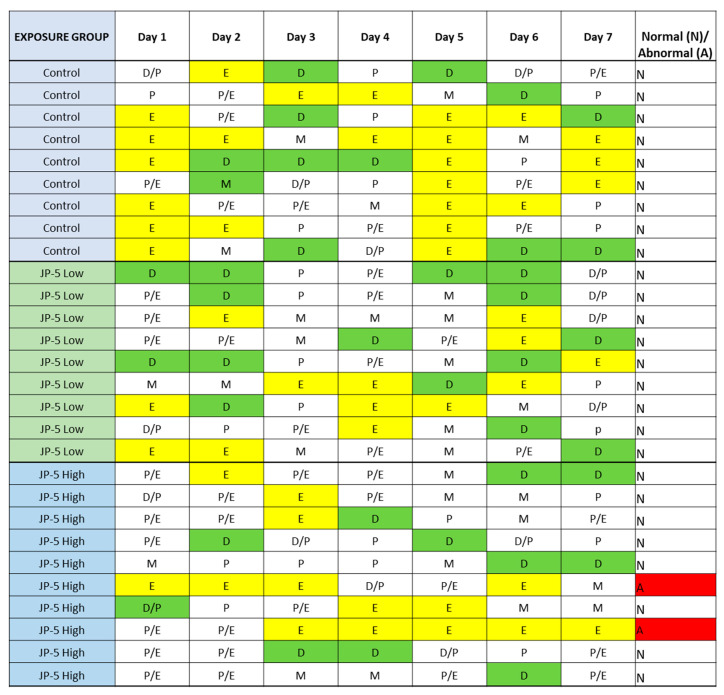
**Estrous cycles from vaginal cytology.** Analyses were performed as delineated in Materials and Methods. Abnormal cycles of three or more days of estrus phase are noted with “A” in red for abnormal; 100% of Control animals with no jet fuel exposure were found to have normal estrous cycles, as were 100% of animals with “low” 750 mg/kg body weight) jet fuel exposure levels. Animals with “high” (2000 mg/kg body weight) jet fuel exposure levels presented with 20% abnormal estrous cycles. The results indicate a potential trend for jet fuel disruption of normal estrous cycles in rats, but the sample size was not enough to confer statistical significance to the results. Phases noted by single letters are: E = Estrus (yellow); D = Diestrus (Green); M = Metestrus; P = Proestrus.

There were no effects on the left or right ovary weights for females exposed to JP-5, as shown in Figure 10 (*p* > 0.05, n = 9 control, JP-5 750 mg/kg, n = 10 JP-5 2000 mg/kg, one-way ANOVA). There were, however, disruptions in estrous cycles noted with JP-5 2000 mg/kg exposure for two of the 10 females, as shown in Figure 11, but the sample size did not confer statistical significance and, furthermore, the timeframe should be longer, but we include this preliminary data based on the fact that the methodology of counting three or more days in a row of estrus as an abnormal cycle would establish the two cycles noted as abnormal in Figure 11, even if the observation time was increased to two weeks. 

**Figure 12:**
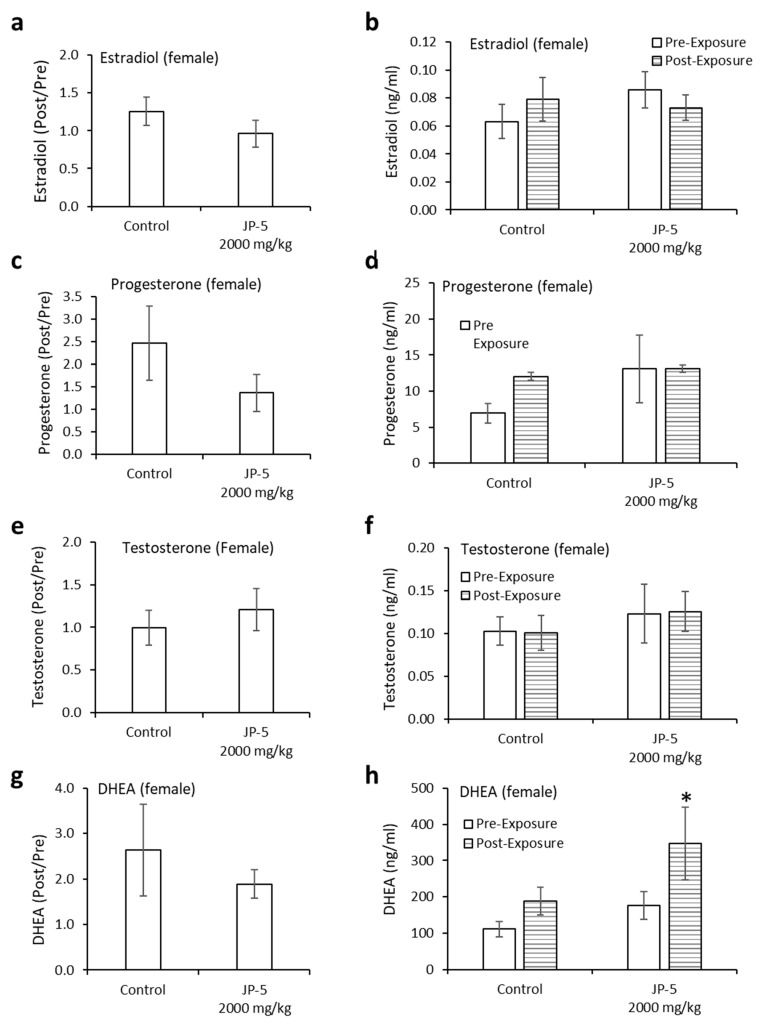
**Hormone levels for female rats following JP-5 exposure.** Hormone levels of female rats in the control and JP-5 High groups were measured as described in Methods. (**a**) There was no statistically significant effect of JP-5 exposure on estradiol levels when data are expressed as the ratio of post-exposure to pre-exposure values (*p* = 0.14, unpaired *t*-test, n = 8). (**b**) There was also no statistically significant effect of JP-5 exposure when post-exposure estradiol data were compared to their respective pre-exposure values (*p* > 0.05, paired *t*-test n = 8). (**c**) There was no statistically significant effect of JP-5 exposure on progesterone levels when data are expressed as the ratio of post-exposure to pre-exposure values (*p* = 0.27, unpaired *t*-test, n = 8 JP-5 High, n = 9 control). (**d**) There was also no statistically significant effect of JP-5 exposure when post-exposure progesterone data were compared to their respective pre-exposure values (*p* > 0.05, paired *t*-test, n = 8 JP-5 High, n = 9 control). (**e**) There was no statistically significant effect of JP-5 exposure on testosterone levels when data are expressed as the ratio of post-exposure to pre-exposure values (*p* = 0.35, unpaired *t*-test, n = 7). (**f**) There was also no statistically significant effect of JP-5 exposure when post-exposure testosterone data were compared to their respective pre-exposure values (*p* > 0.05, paired *t*-test n = 7). (**g**) There was no statistically significant effect of JP-5 exposure on DHEA levels when data are expressed as the ratio of post-exposure to pre-exposure values (*p* = 0.50, unpaired *t*-test, n = 9). (**h**) There was a statistically significant increase in DHEA levels post-JP-5 exposure at 2K mg/mL compared to the baseline DHEA levels pre-exposure (* *p* = 0.049, paired *t*-test, n = 9). Data are presented as mean *+/−* SEM.

Neither estradiol nor progesterone levels were significantly affected by JP-5 exposure (Figure 12a–d). There were trending non-significant reductions in the estradiol level in female rats compared to the control when the data were analyzed as ratios of post- to pre-exposure values (*p* = 0.14, two-tailed, unpaired *t*-test, n = 8) (Figure 12a). A comparison of pre-exposure with post-exposure levels within exposure groups did not show statistically significant differences when analyzed using a two-tailed paired *t*-test (*p* > 0.05). However, a slight trending increase was noted in the control post-exposure average compared to the control pre-exposure level, whereas there was a sight trending decrease in the JP-5 2000 mg/kg post-exposure average compared to the JP-5 2000 mg/kg pre-exposure level (Figure 12b).

There were trending non-significant reductions in the progesterone levels in female rats compared to the control when data were analyzed as ratios of post- to pre-exposure values (*p* = 0.27, two-tailed, unpaired *t*-test, n = 8 JP-5, n = 9 control) (Figure 12c). A comparison of pre-exposure with post-exposure levels within exposure groups did not show statistically significant differences when analyzed using a two-tailed paired *t*-test (*p* > 0.05, paired *t*-test) (Figure 12d).

There were very slight trending increases in the testosterone levels in female rats compared to the control when data were analyzed as ratios of post- to pre-exposure levels (*p* = 0.35, two-tailed, unpaired *t*-test, n = 7) (Figure 12e). There was no noticeable difference in the averaged post-exposure value compared to the pre-exposure value of testosterone levels in the control or JP-5 2000 mg/kg groups (*p* > 0.05) (Figure 12f).

There were slight trending increases in the DHEA levels in female rats compared to the control when data were analyzed as ratios of post- to pre-exposure levels (*p* = 0.50, two-tailed, unpaired *t*-test, n = 9) (Figure 12g). Interestingly, there was an increase in the averaged post-exposure value of DHEA compared to the pre-exposure value in the JP-5 2000 mg/kg group (*p* = 0.049), a difference that was not present in the control group (*p* = 0.1) (Figure 12h).

### 3.5. Mating Efficiency

Due to the attrition of animals that was not attributable to the ingestion of JP-5, the mating groups were not uniform. The control group consisted of 25 pairs, of which 23 successfully mated and produced offspring, for a mating success rate of 92%. The JP-5 at 750 mg/kg group had 24 pairs, of which 22 successfully mated and produced offspring, for a mating success rate of 91.7%. The 2000 mg/kg exposure group consisted of 18 pairs, of which 16 pairs successfully mated and produced offspring, for a mating success rate of 88.9%. 

### 3.6. Offspring Effects

**Figure 13:**
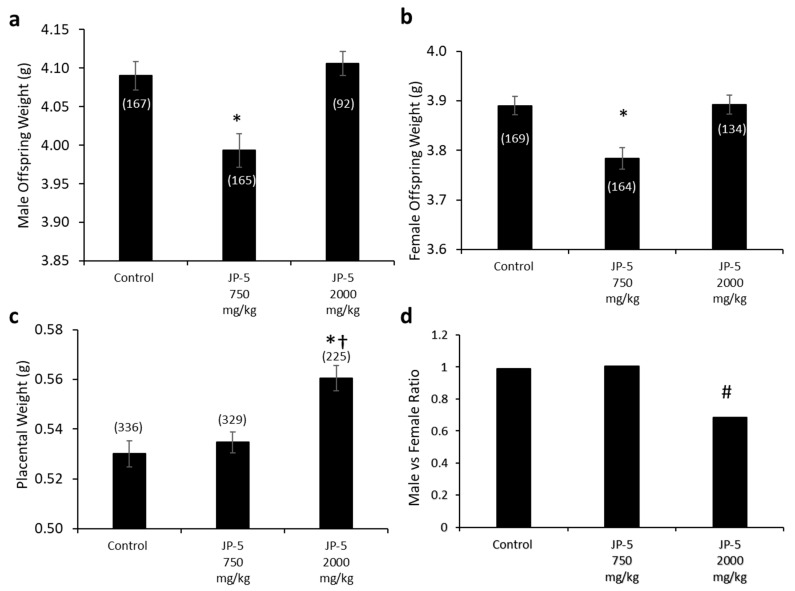
**Fetal data for offspring born to rats exposed to jet fuel before mating, with the dams continuing exposure through pregnancy.** (**a**) Average fetal body weight for male offspring was significantly decreased for the JP-5 750 mg/kg group compared to control (* *p* = 0.035). (**b**) Average fetal body weight for female offspring was significantly decreased for the JP-5 750 mg/kg group compared to control (* *p* = 0.008). (**c**) There was a significant increase in average placental weight for rats exposed to 2000 mg/kg of JP-5 compared to control (* *p* < 0.001) or JP-5 Low group († *p* = 0.011). For a-c, numbers in parentheses represent n values and data are presented as mean *+/−* SEM. (**d**) The male-to-female offspring ratio was significantly reduced in dams that were exposed to 2000 mg/kg of JP-5 (# *p* = 0.003).

Fetal evaluations were performed on GD 20 in accordance with a study by Cooper and Mattie (1996) and as described in the Materials and Methods. The weights of offspring were significantly lower following JP-5 750 mg/kg exposure for both males (Figure 13a) and females (Figure 13b). The male and female offspring weight data failed normality, and thus, the non-parametric Kruskal–Wallis one-way ANOVA was used to assess statistical significance. The Kruskal–Wallis analysis of the male offspring data was significant across experimental groups, with H = 9.990 (df = 2), *p* = 0.007 (n = 92–167 as indicated in the Figure 13a graph). Multiple comparison using Dunn’s method yielded significantly lower male offspring weights from the JP-5 750 mg/kg group compared to the control group, with Q = 2.525, *p* = 0.035. The Kruskal–Wallis analysis of female offspring data revealed a significant difference across experimental groups, with H = 9.961 (df = 2), *p* = 0.007 (n = 134–169 as indicated in the Figure 13b graph). Multiple comparison using Dunn’s method yielded significantly lower male offspring weights from the JP-5 750 mg/kg group compared to the control group, with Q = 2.999, *p* = 0.008.

The numbers of pups per litter across the exposure groups were very uniform. There were 23 total litters for the control group, with a mean litter size of 15 pups per litter and a range of 10–21. There were 22 total litters included in the JP-5 750 mg/kg evaluations, with a mean litter size of 15 pups per litter and a range of 13–20. There were 16 litters included in the JP-5 at 2000 mg/kg group, with a mean litter size of 15 pups per litter and a range of 12–18. 

Interestingly, the fetal weights trended back to the same as the controls for the JP-5 2000 mg/kg-exposed rats for both males (Figure 13a) and females (Figure 13b); the reason for the seeming paradox can be discerned from the increased placental weight noted for JP-5 2000 mg/kg exposure (Figure 13c). The combined male and female placental weight data also failed normality and were statistically analyzed using a Kruskal–Wallis one-way ANOVA, yielding significant difference across groups, with H = 18.262 (df = 2), *p* < 0.001 (n = 225–336). Multiple comparison using Dunn’s method yielded significantly greater placental weights from the JP-5 2000 mg/kg group compared to the control (Q = 4.247, *p* < 0.001) or JP-5 750 mg/kg groups (Q = 2.912, *p* = 0.011). Estrogen has a positive effect on vascularization and the growth of both the placenta and the fetus [20] and the increased placental weight noted could be the result of increased estrogenic compound exposure, which would point to JP-5 acting as an endocrine disruptor, specifically as an estrogen mimic. The seeming paradox of fetal weight loss with a lower exposure level of JP-5 while the weight is increased with higher exposure can be attributed to the larger placenta, which would provide more nutrients to the offspring of the JP-5 2000 mg/kg-exposed dams. 

Figure 13d shows the male-to-female ratio of offspring for the exposure groups, with approximately the expected 1:1 value for both the control and JP-5 750 mg/kg exposure groups, with the control group having 167 male and 168 female fetuses while the JP-5 750 mg/kg group had 165 male and 164 female fetuses. The JP-5 2000 mg/kg group, on the other hand, showed an approximately 0.69:1 ratio of males to females, which was found to be a statistically significant ratio difference (*p* = 0.003) using binomial distribution analysis with the assumption that the distribution should be 50% based on the fact that spermatogenesis is a meiotic process that will produce 50% Y and 50% X chromosome-bearing spermatids (Figure 12d). There were fewer dams in the final analysis for the JP-5 2000 mg/kg group (16) compared to the control and JP-5 750 mg/kg groups (20 each), with the fetuses-per-dam ratio being 15.3 for the control group, 15.5 for the JP-5 750 mg/kg group, and 14.1 for the JP-5 2000 mg/kg group. There were 16 resorptions noted in the JP-5 2000 mg/kg group, vs. 11 each in the control and JP-5 750 mg/kg groups, which means that the resorption-to-fetus ratio was 0.07 for the JP-5 2000 mg/kg group, compared to 0.04 for the control and JP-5 750 mg/kg groups. Normalizing the resorption rate for JP-5 to the control rate would mean that the expected resorptions for the JP-5 2000 mg/kg group would be 9.01, suggesting that there were seven resorptions that could potentially be attributed to the effects of JP-5 2000 mg/kg exposure; if all seven of these excess resorptions were assumed to be male, the ratio of male to female fetuses would be 0.74:1, which would still fail the binomial distribution assumption of 1:1, or 50% male, with *p* = 0.013. The importance of the fact that the binomial distribution would still be significantly skewed towards more females than should be expected is that this indicates a mechanism of action that leads to successful fertilization by X chromosome-bearing spermatids, rather than that the JP-5 2000 mg/kg exposure was preferentially toxic to male fetuses, although this preferential toxicity to male fetuses may also be true, but there is no way to know the sex associated with the resorptions.

The fetuses were further inspected for anogenital distance, abnormalities of which can be an indication of endocrine disruption, as well as for gross abnormalities. There were no indications that the JP-5 exposures produced any alterations in anogenital distance. 

### 3.7. In Vitro Activation of Human Estrogen Receptors by Jet Fuels

Due to the potential endocrine-disrupting effects noted for the study described thus far, we tested the ability of JP-5 and other jet fuels to directly activate human hormone receptors, which would be a likely mode of endocrine disruption. The jet fuels selected for testing were JP-5, JP-8, and the Bio-oil-Derived Synthetic Paraffinic Kerosene derived from the camelina plant, HRJ. While the animal study provides some evidence of JP-5 being an estrogen mimic, the structural evaluation of its components provides some indication that it might have a structural similarity to estradiol (Figure 1). We also tested JP-8, a similar U.S. Air Force jet fuel that contains similar constituents. The compound β-sitosterol is a known estrogen receptor activator [13], and since it is a component of camelina plant oil, it seemed possible that it could survive refinement into jet fuel, which turns camelina oil into HRJ fuel (Figure 1). In addition to the human estrogen receptor assays, an assay was performed with the human androgen receptor and the human glucocorticoid receptor to test the hormone receptor specificity of the jet fuels. Only the estrogen receptor evaluations showed any sign of being activated by jet fuels, with the camelina-derived synthetic HRJ showing no indication of activating the steroid receptors.

**Figure 14:**
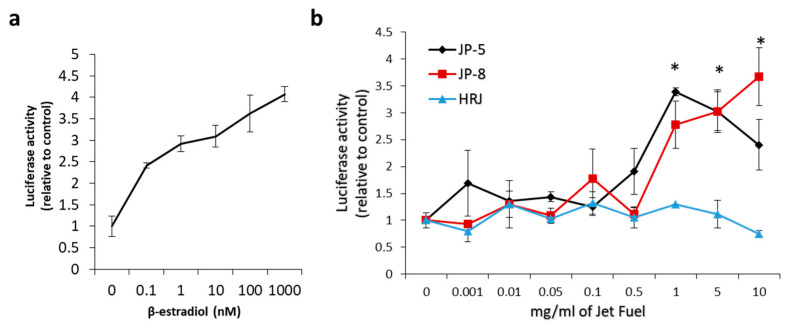
**Effects of in vitro jet fuel exposure on human estrogen receptors.** (**a**) 17β-estradiol activation of the estrogen receptor. Results show an expected activation of the estrogen receptor. A human estrogen receptor mammalian expression plasmid, a β-galactosidase expression plasmid, and a luciferase reporter construct plasmid with an estrogen receptor response element were transfected into HEK293 cells and analyzed for ligand stimulation of receptor activities. 17β-estradiol is an endogenous steroid known to activate the human estrogen receptor. Luciferase activity is expressed as fold activation vs. zero concentration control. Results were normalized using the constitutively active β-galactosidase expression plasmid that shows cell viability. Results were also shown to be dependent on the co-transfected estrogen receptor plasmid as a further control. Data presented as mean *+/−* SEM, with n = 3. (**b**) Results show that JP-5 (black) and JP-8 jet fuel are capable of activating the human estrogen receptor. Data shown are normalized to control values (0 mg/mL). Statistically significant activation is indicated by an asterisk above the bar in the graph for the JP-5 and JP-8 data (* *p* < 0.05, one-way ANOVA, Holm–Sidak method vs. 0 mg/mL of JP-5 or JP-8). Data presented as mean *+/−* SEM, with n = 3 for each jet fuel tested (JP-5, JP-8, HRJ).

Figure 14a shows the concentration response curve that resulted from the addition of increasing concentrations of 17β-estradiol, which is a known agonist of the human estrogen receptor-α. 17β-estradiol was used in separate experiments at concentrations of 0, 0.1 nm, 1 nm, 10 nm, 100 nm, and 1000 nm in the mammalian cell co-transfection assay using HEK293 cells as described in the Materials and Methods. Luciferase activity is expressed as fold activation vs. zero concentration control. The results were normalized using the constitutively active β-galactosidase expression plasmid that shows cell viability. These results show that the test system was working as expected.

To test the ability of jet fuels to modulate human estrogen receptor-α activity, JP-8, JP-5, or HRJ were tested at concentrations of 0, 0.1, 1, 5, and 10 mg/mL. The results showed that JP-5 and JP-8 were able to positively regulate the human estrogen receptor with a statistically significant level of activity reached, beginning at a concentration of 1 mg/mL (Figure 14b). An analysis of the JP-5 data revealed a significant difference across concentration groups, with F(8, 18) = 6.403, *p* < 0.001, n = 3. Multiple comparison using the Holm–Sidak method yielded a significant activation of ER in the 1 (*p* < 0.001), 5 (*p* = 0.003), and 10 (*p* = 0.043) mg/mL groups when compared to 0 mg/mL. An analysis of the JP-8 data revealed a significant difference across concentration groups, with F(8, 18) = 8.125, *p* < 0.001, n = 3, with significant activation of the estrogen receptor in the 1 (*p* < 0.001), 5 (*p* = 0.007), and 10 (*p* = 0.016) mg/mL groups when compared to 0 mg/mL. An analysis of the HRJ data did not reveal significant activation of the estrogen receptor at the concentrations tested, with F(8,18) = 1.471, *p* = 0.235, n = 3. 

The androgen receptor was shown to be responsive to testosterone as expected, but exposures to jet fuels showed no statistically significant effects in the assay. Likewise, the dexamethasone results showed that the assay was working as expected for the glucocorticoid receptor, but the assay showed no indication that jet fuels modulated glucocorticoid activity.

## 4. Discussion

The importance of understanding the effects of ingested jet fuel has been brought into the spotlight by recent events where accidental spills led to the presence of jet fuel in drinking water. This study used relatively high concentrations of JP-5 jet fuel to look at potential endocrine disruption in adult rats, effects on mating and fertility, and effects on fetuses due to parental exposure. There were some indications of gastric distress, which would be expected from ingesting high concentrations of jet fuel, which manifested as a significantly lower daily food intake for males (Figure 3), while females showed a significantly lower daily food intake prior to pregnancy (Figure 6a), but that trend was reversed during pregnancy, with the female rats then showing higher food intake when exposed to JP-5 (Figure 6b). For the males, this diminished food intake may have manifested as lower overall body weights for rats exposed to JP-5 (Figure 2a), but the percentage weight gain was similar for all rats including controls (Figure 2b). For females, only those rats exposed to JP-5 at the higher concentration used in this study were found to have significantly lower final weights pre-pregnancy in both absolute weight (Figure 4a) and percentage weight gained (Figure 4b), while the pregnant rats showed no difference in percentage weight gained (Figure 5). The fact that the pregnant females ate significantly more food with JP-5 exposure may indicate some compensatory eating during pregnancy for being malnourished before mating, but the results indicate nothing beyond the expected gastric distress that might be expected from oral exposure to jet fuel.

This study found no effects on male reproduction in terms of mating behavior. We attribute the noted differences in the weights of testes and epididymes when compared to body weight with JP-5 exposure (Figure 7b,d) to the diminished body weight gain of the adult rats, but this observation is included since size alterations of these organs would be noteworthy if attributable to exposure. There were no observations of gross abnormalities. There were no negative effects on sperm count (Figure 8a) or motility (Figure 8b–d), but there was a trending, non-significant increase in path linearity for sperm (Figure 8e) which is not normally looked at as an adverse effect, since linearity has a relationship with fertilization success. In context with other findings in this study that indicate JP-5 as being capable of regulating estrogen receptor activity, as discussed further below, it should be noted that if this increased path linearity for sperm exposed to JP-5 holds true, a possible explanation could be that estrogen receptors present in sperm have been shown to regulate motility [21], so while attributing the observation to JP-5 exposure is only a remote possibility, it could warrant further study.

Estradiol, a hormone that is an endogenous ligand of the estrogen receptor, is associated with normal sexual behavior in both males and females. Figure 9b shows how post-exposure estradiol levels were significantly lower for male rats following more than 9 weeks of JP-5 exposure, while there were no statistical differences comparing post- to pre-exposure estradiol levels in control rats. Since the rats mated successfully, there were no noted effects of these altered levels, but these diminished estradiol levels could indicate the possibility of negative feedback to estradiol production due to JP-5 exposure. While female rats did not show a significant diminishment of estradiol levels over the course of a shorter period of exposure than the males (3 weeks for females vs. more than 9 weeks for males), there was a JP-5-associated “trend” in the diminishment of estradiol levels comparing post-exposure to pre-exposure animals (Figure 12b). These estradiol levels provide some evidence of endocrine disruption, with the possibility of being tied to JP-5 serving as an estrogen mimic. This study did not entertain the idea that some fuel constituents may, in fact, be hormone receptor antagonists.

Progesterone is a hormone that is normally associated with female sexual traits and pregnancy, but it is also important for normal male sexuality, and increased levels of progesterone may be associated with sexual activity in males [22]; therefore, the trending increase in progesterone levels for male rats in the control group noted in this study should be looked at from the perspective that the rats had been involved in mating prior to the final blood collection for hormone analyses, which should explain the increase in progesterone levels noted in the control group (Figure 9d). The fact that the JP-5-exposed groups did not show a similar increase in progesterone levels despite the same mating circumstances prior to the hormone analyses makes the diminishment in progesterone levels with JP-5 exposure all the more worthy of note, even if it is not statistically significant. Female rats also showed a trending non-significant reduction in progesterone levels when viewed as post- vs. pre-exposure levels (Figure 12d). These observations provide further evidence of endocrine disruption due to JP-5 exposure.

Testosterone and DHEA are androgenic hormones strongly associated with male sexual characteristics and behavior, but they are also important in females. There were no observed differences in the androgenic hormones evaluated in this study for male rats, but the females in this study did show a statistically significant increase in DHEA levels when comparing post- to pre-exposure with JP-5. While these changes in DHEA levels are slight, it is possible they are due to the endocrine disruption properties of JP-5 and warrant further study.

The rat estrous cycle is usually 4–5 days in duration and is divided into four phases: estrus, metestrus, diestrus, and proestrus [23]. Remaining in the estrus phase for 3 days or longer is considered an aberrant cycle [24]. Both estrogen and progesterone are commonly associated with normal estrous cycles, and perturbations of levels of these hormones could lead to altered estrous cycles. As shown in Figure 11, JP-5 at the 2000 mg/kg body weight exposure level showed a trend towards aberrancy with 20% abnormal cycles, providing further support for the endocrine-disrupting potential of JP-5.

Estrogen plays a positive role in both placental and fetal angiogenesis and is associated with the vascularization and growth of the placenta and fetus [20]. JP-5 exposure at 750 mg/kg body weight exhibits a toxic effect that resulted in significantly diminished fetal weights for both males (Figure 13a) and females (Figure 13b). Paradoxically, this diminished fetal weight for both sexes was not present at the higher concentration of JP-5 used (Figure 13a,b). The significantly larger placenta sizes shown in Figure 13c for dams exposed to the higher level of JP-5 could be an explanation for this observation and would lend further support to JP-5 acting as an endocrine disruptor, particularly acting as an estrogen mimic. 

The increased number of female offspring with the higher concentration of JP-5 is not explained by the number of excess fetal resorptions, since even if all of these resorptions were male, a fact we cannot know, there would still be significantly more females. It is difficult to provide an explanation without further study, but one possibility to consider is that the JP-5 exposure provides an advantage in fertilization success to X chromosome-bearing spermatids in either survival or motility. There is also the possibility that JP-5 is more toxic to the developing male fetus and therefore caused more fetal resorptions for males. The fact that there were only resorptions and not non-viable fetuses present would perhaps indicate that the problems targeting male offspring occur in early development or prior to fertilization. 

The most noteworthy finding of this study is the observation of estrogen receptor activation by the jet fuels JP-5 and JP-8 using an in vitro activation assay with human estrogen receptor and human embryonic kidney cells, with statistically significant activation compared to controls beginning at levels of 1 mg/mL (Figure 14b). The cycloparaffin and aromatic structures present in both JP-5 and JP-8 have some similarity to the shapes of estrogenic and androgenic compounds, as shown in Figure 1. The alternative jet fuel (HRJ) used in the steroid receptor assay is derived from the camelina plant, which is known to produce a phytoestrogen with a demonstrated ability to regulate the estrogen receptor [13], but the HRJ jet fuel showed no signs of estrogen receptor modulation in our study (Figure 14b).

Mechanistically, endocrine disruptors can act in several ways, including nuclear receptor binding, the disruption of hormone signaling pathways, interfering with hormone synthesis, interference with hormone-binding proteins, or interference with the expression of hormone receptors [25]. Binding to a nuclear hormone receptor is the mode of action expected from a compound that resembles an endogenous hormone, but multiple routes could be possible, especially with a substance such as JP-5 that has multiple constituents. The observation of the upregulation of human estrogen activity using an in vitro assay in this study provides evidence that JP-5 and JP-8 can act as ligands for the receptor in the same way that estrogens do, but it is possible that the indications of JP-5 acting as an endocrine disruptor under the conditions studied are due to the jet fuel acting through other pathways instead of, or in addition to, estrogen receptor binding. The outcomes of endocrine disruptor exposure can be difficult to predict even with an expected mechanism of action, since the effects of perturbations of hormonal actions are complex: testosterone is converted to estradiol by the same enzyme in both males and females, males require estrogens for normal sexual function, and females normally have higher levels of serum androgen levels than estrogens, except during certain estrous cycle phases, although these higher androgen levels could simply be reflective of the need for estrogen, for which androgens are precursors [26]. This complexity makes it difficult to surmise pathways of action based on observations of hormone levels or effects associated with hormones, but perturbations can infer endocrine disruption that may require further clarification through studies designed to elucidate the observations. 

## 5. Conclusions

While this study used relatively high concentrations of JP-5 to elucidate the potential for endocrine disruption, further studies would be needed with more physiologically and environmentally relevant exposure levels to gain a proper understanding of the potential dangers of JP-5 exposure to the endocrine system. The results do, however, provide strong support to the notion that JP-5 can act as an endocrine disruptor, with the structural similarity of cyclic carbon components of JP-5 to endogenous human steroids as the most likely mechanism of action, with these components acting as mimics of steroids with potential for modulating steroid receptors or providing negative feedback that could cause the body to decrease the production of certain steroids. The finding that JP-5 and JP-8 can both activate the estrogen receptor using an in vitro assay in human cells with a human estrogen receptor lends very strong support to the notion that these jet fuels can function as estrogen mimics. While these cyclic carbon components may very well be confirmed to be steroid mimics in the body, it should also be kept in mind that it is very possible that environmentally relevant exposures to these jet fuels might reasonably be considered safe, even if the components can represent a health hazard at high exposure levels. 

Further characterization is needed to help resolve potential hazards associated with the ingestion of these jet fuels. We currently have ongoing studies that will further investigate the role of estrogen receptors following oral exposure to JP-5. We will also evaluate whether prolonged repeated exposure to lower doses of JP-5 can have detectable effects on neurological and endocrinological endpoints in offspring.

## Data Availability

The data that support the findings of this study are not openly available due to U.S. government clearance restrictions and are available from the corresponding author, WRH (william.howard.35@us.af.mil), upon reasonable request. Each request to the corresponding author will be handled on a case-by-case basis in compliance with any and all U.S. government security requirements. Data are stored under controlled access at the Naval Medical Research Unit Dayton.
